# A Primary Care–Based Weight Navigation Program

**DOI:** 10.1001/jamanetworkopen.2024.12192

**Published:** 2024-05-21

**Authors:** Dina H. Griauzde, Cassie D. Turner, Amal Othman, Lauren Oshman, Jonathan Gabison, Patricia K. Arizaca-Dileo, Eric Walford, James Henderson, Deena Beckius, Joyce M. Lee, Eli W. Carter, Chris Dallas, Kathyrn Herrera-Theut, Caroline R. Richardson, Jeffrey T. Kullgren, Gretchen Piatt, Michele Heisler, Andrew Kraftson

**Affiliations:** 1 Department of Internal Medicine, University of Michigan Medical School, Ann Arbor; 2 VA Ann Arbor Healthcare System, Ann Arbor, Michigan; 3 University of Michigan Institute for Healthcare Policy and Innovation, Ann Arbor; 4 Department of Learning Health Sciences, University of Michigan Medical School, Ann Arbor; 5 Department of Family Medicine, University of Michigan Medical School, Ann Arbor; 6 University of Michigan Elizabeth Weiser Caswell Diabetes Institute, Ann Arbor; 7 Department of Pediatrics, University of Michigan Medical School, Ann Arbor; 8 Department of Family Medicine, The Warren Alpert Medical School of Brown University and Care New England, Providence, Rhode Island; 9 Department of Health Management and Policy, University of Michigan School of Public Health, Ann Arbor; 10 Department of Health Behavior and Health Education, University of Michigan School of Public Health, Ann Arbor

## Abstract

**Importance:**

Evidence-based weight management treatments (WMTs) are underused; strategies are needed to increase WMT use and patients' weight loss.

**Objective:**

To evaluate the association of a primary care–based weight navigation program (WNP) with WMT use and weight loss.

**Design, Setting, and Participants:**

This cohort study comprised a retrospective evaluation of a quality improvement program conducted from October 1, 2020, to September 30, 2021. Data analysis was performed from August 2, 2022, to March 7, 2024. Adults with obesity and 1 or more weight-related condition from intervention and control sites in a large academic health system in the Midwestern US were propensity matched on sociodemographic and clinical factors.

**Exposure:**

WNP, in which American Board of Obesity Medicine–certified primary care physicians offered weight-focused visits and guided patients’ selection of preference-sensitive WMTs.

**Main Outcomes and Measures:**

Primary outcomes were feasibility measures, including rates of referral to and engagement in the WNP. Secondary outcomes were mean weight loss, percentage of patients achieving 5% or more and 10% or more weight loss, referral to WMTs, and number of antiobesity medication prescriptions at 12 months.

**Results:**

Of 264 patients, 181 (68.6%) were female and mean (SD) age was 49.5 (13.0) years; there were no significant differences in demographic characteristics between WNP patients (n = 132) and matched controls (n = 132). Of 1159 WNP-eligible patients, 219 (18.9%) were referred to the WNP and 132 (11.4%) completed a visit. In a difference-in-differences analysis, WNP patients lost 4.9 kg more than matched controls (95% CI, 2.11-7.76; *P* < .001), had 4.4% greater weight loss (95% CI, 2.2%-6.4%; *P* < .001), and were more likely to achieve 5% or more weight loss (odds ratio [OR], 2.90; 95% CI, 1.54-5.58); average marginal effects, 21.2%; 95% CI, 8.8%-33.6%) and 10% or more weight loss (OR, 7.19; 95% CI, 2.55-25.9; average marginal effects, 17.4%; 95% CI, 8.7%-26.2%). Patients in the WNP group were referred at higher rates to WMTs, including bariatric surgery (18.9% vs 9.1%; *P* = .02), a low-calorie meal replacement program (16.7% vs 3.8%; *P* < .001), and a Mediterranean-style diet and activity program (10.6% vs 1.5%; *P* = .002). There were no between-group differences in antiobesity medication prescribing.

**Conclusions and Relevance:**

The findings of this cohort study suggest that WNP is feasible and associated with greater WMT use and weight loss than matched controls. The WNP warrants evaluation in a large-scale trial.

## Key Points

**Question:**

Can a primary care–based weight navigation program (WNP) support weight loss among patients with obesity through weight-focused consultation visits with obesity medicine experts and preference-sensitive use of weight management treatments?

**Findings:**

In this cohort study including 264 patients, WNP patients (n = 132), compared with matched controls (n = 132) were referred at higher rates to health system weight management treatments. In addition, patients in the WNP lost significantly more weight (−4.4% vs −0.1%) and had greater odds of achieving 5% or more and 10% or more weight loss compared with controls.

**Meaning:**

The findings of this study suggest that the WNP model offers a promising approach to improve treatment and outcomes for patients with obesity and warrants further evaluation in a large-scale clinical trial.

## Teaser

This cohort study examines the use of a weight navigation program comprising primary care physicians offering weight-focused visits and guidance on patients’ selection of treatment.

## Introduction

In the US, obesity is a leading cause of morbidity, mortality, and health care spending.^1,2,3,4^ Among individuals with obesity, as little as 5% weight loss can improve cardiometabolic health,^5^ prevent progression to type 2 diabetes (T2D),^6^ and reduce health care expenditures.^7^ Despite the availability of multiple weight management treatments (WMTs), most are severely underused.^8^ For example, among eligible individuals, less than 5% are referred to diabetes prevention programs,^9^ less than 4% are currently prescribed antiobesity medications (AOMs),^10^ and less than 1% undergo bariatric surgery.^11^ Multiple barriers impede the use of successful WMTs by primary care practitioners (PCPs), including inadequate training in obesity medicine and brief clinic visits with competing clinical priorities.^12,13,14^

With increasing obesity rates,^15^ novel strategies are needed to overcome treatment barriers. The American Board of Obesity Medicine (ABOM) continuing medical education pathway can address PCPs knowledge gaps.^16,17^ Currently, there are approximately 8200 ABOM diplomates, most (65%) are PCPs, and evidence suggests that they use the full range of WMTs,^18^ often in the context of weight-focused visits.^19,20,21^ Yet, the overall number of diplomates remains low relative to the many patients who may desire and benefit from preference-sensitive use of WMTs.^22,23^ Moreover, little is known about how to incorporate diplomates into primary care teams to enhance the reach of their expertise.

To address this gap, our team developed and tested a primary care–based weight navigation program (WNP).^24^ The WNP draws on principles from the Collaborative Chronic Care Model^25,26,27^ and aims to integrate ABOM diplomates into primary care teams. Diplomates offer weight-focused consultation visits and support patients’ selection of preference-sensitive WMTs. The objectives of this study were to evaluate WNP feasibility and association with WMT use and weight loss. We hypothesized that patients in the WNP would have higher rates of WMT use and greater weight loss than matched controls from another primary care clinic.

## Methods

### Study Design

This was a retrospective evaluation of the WNP using matched cohort analysis to evaluate the program’s first year (October 1, 2020, to September 30, 2021). Data analysis was performed from August 2, 2022, to March 7, 2024. The study was approved by the University of Michigan Institutional Review Board. Patients did not provide informed consent for this secondary analysis of electronic health record (EHR) data. Findings are reported using the Strengthening the Reporting of Observational Studies in Epidemiology (STROBE) reporting guideline.

### Study Setting

The WNP was implemented in 1 primary care clinic affiliated with a large, academic medical center. Matched controls were patients from a neighboring primary care clinic affiliated with the same medical center. The medical center offers various WMTs, including (1) primary care–based nutrition counseling with a registered dietitian, (2) a lifestyle change program focused on Mediterranean-style eating and physical activity, (3) a medically supervised very low-calorie meal replacement program, and (4) bariatric surgery. The EHR also includes a referral to community-based diabetes prevention programs.

### Participants

Eligibility for the WNP included (1) receiving primary care at the intervention clinic, (2) body mass index (BMI) of 30 or more (calculated as weight in kilograms divided by height in meters squared), and (3) 1 or more weight-related chronic condition, including T2D, hypertension, hyperlipidemia, metabolic dysfunction–associated fatty liver disease, or obstructive sleep apnea. Patients at the neighboring clinic with BMI of 30 or more and 1 or more weight-related chronic condition were eligible controls.

### Intervention

The WNP was previously described.^24^ Briefly, PCPs at the intervention clinic were informed of WNP eligibility criteria through email and brief presentations. Primary care practitioners could refer patients using an EHR order that prompted ordering of cardiometabolic laboratory tests (eg, hemoglobin A_1c_, lipid levels). Once scheduled, patients received an EHR-based weight history questionnaire.

Weight navigation program physicians are ABOM diplomates who practice primary care and offer weight-focused visits for approximately 4 hours per week. Diplomates obtained in-depth knowledge of local WMTs, including patient eligibility criteria, insurance coverage, and out-of-pocket costs, by observing key personnel in all health system weight management programs; this experience consisted of 40 hours of observation over 16 weeks.

During the initial 60-minute WNP visit, the diplomate reviewed with patients their weight and medical histories, laboratory data, and weight loss goals. Obesogenic conditions and medications were identified and addressed as needed. Diplomates informed patients of all WMTs for which they were eligible and potential costs based on patients’ insurance type. Diplomates guided patients’ treatment selection and used structured note templates to document the initial plan and potential alternatives that could be offered by PCPs, as needed, based on initial treatment response.

The diplomate placed initial referral orders. We intended for diplomates to guide PCPs’ AOM prescribing by documenting the recommended AOM, its titration schedule, and indications to stop, continue, or escalate the dose. However, PCPs requested the diplomate to carry out these steps.

A subset of WNP patients (n = 52) was invited to participate in a single-arm pilot study including weight reporting by text message; 27 patients enrolled. This protocol was previously described^24^ and will be fully evaluated separately.

Visits were billed using the 99215 Evaluation and Management code for a 60-minute visit. The 99417 Evaluation and Management code was added if the PCP spent an additional 15 minutes on same-day documentation.

### Outcome Measures

Primary outcomes included measures of WNP feasibility,^28^ including rates of WNP referral (ie, the number of WNP-referred patients divided by the number of WNP-eligible patients), and uptake (ie, the number of patients who completed a WNP visit divided by the number referred). Secondary outcomes included change in weight comprising evaluation of mean weight change in kilograms, percent weight loss, and achievement of 5% or more and 10% or more weight loss. For WNP patients, weight data were abstracted from the EHR at baseline, defined as the date of the first WNP appointment. For matched controls, the baseline weight was the first documented weight within the 1-year study period. The 12-month follow-up measurement for both WNP participants and matched controls was the weight closest to 12 months after baseline. Patients without follow-up weight data within 12 months ±90 days were excluded from weight change analyses.

Referrals to WMTs included referrals to any of the following health system programs: (1) primary care–based nutrition counseling with a registered dietitian, (2) a medically supervised very low-calorie meal replacement program, (3) a lifestyle change program focused on Mediterranean-style eating and physical activity, and/or (4) bariatric surgery. We also evaluated referral to community diabetes prevention programs.

Patients’ engagement with health system WMTs was defined as having 1 or more completed visit during the study period with the WMT to which they were referred. Among patients referred to bariatric surgery, we evaluated the number of completed surgeries. We were unable to assess patients’ engagement with community diabetes prevention programs, as programs do not report this information to referring PCPs.

Prescriptions for AOMs were defined as at least 1 order for an AOM with US Food and Drug Administration approval during the study period, including orlistat, liraglutide, naltrexone and bupropion, phentermine and topiramate, and phentermine. To account for off-label prescribing, we included overlapping exposures for either phentermine and topiramate or naltrexone and bupropion. In a post hoc analysis, we examined prescriptions for glucagonlike peptide-1 receptor agonists approved for T2D management during the study period, as some are now approved to treat obesity in patients without T2D (eg, semaglutide).

### Matched Cohort Analysis

#### Baseline Characteristics

Using EHR data, we identified patients meeting WNP eligibility criteria at intervention and control sites. We extracted from the EHR patients’ height, weight, BMI, diagnoses for weight-related conditions, and demographic variables, including age, sex, and self-reported race and ethnicity from a list of predefined choices, that were included to describe the patient population and used analytically for matching. We also extracted socioeconomic variables, including insurance status and National Neighborhood Data Archive neighborhood socioeconomic characteristics (ie, proportion of households in the neighborhood below the poverty line and proportion of households that have household income greater than $75 000).^29^ For National Neighborhood Data Archive geolocation data, each patient’s most recent address was mapped to a US census block group and tract, which was then mapped to identify the percentage of households in the patient’s census block group and tract. We imputed missing National Neighborhood Data Archive values using the median.

#### Propensity Matching

Among WNP-eligible patients at the intervention site, we modeled the propensity for WNP participation using logistic regression with independent variables of age, sex, race and ethnicity, baseline BMI, Medicare insurance status, weight-related conditions, and percentage of households in the neighborhood with household income greater than $75 000 per year (eTable 1 in Supplement 1). We included the proportion of households with income greater than $75 000 due to its association with WNP referral and included other variables a priori. We propensity matched WNP patients 1:1 to controls. We assessed balance by calculating standardized mean differences (SMDs) for propensity model variables.^30^

### Statistical Analysis

We computed descriptive statistics for baseline characteristics, including means, medians, minimums, and maximums for continuous variables and counts and proportions for categorical variables. We calculated *P* values for baseline characteristics using analysis of variance for continuous variables and χ^2^ tests for categorical variables. Among WNP-eligible patients at the pilot site, we assessed differences using multivariate logistic regression between patients referred and not referred to WNP and WNP-referred patients who did and did not complete a visit.

Among the matched cohort, we computed rates of referral to WMTs occurring between baseline and 12 months. We used logistic regression to compare WNP vs control patients on the probability of achieving 5% or more and 10% or more weight change and the probability of being referred to WMTs or prescribed AOMs. For outcomes with fewer than 5 occurrences in either arm, we used Firth bias-reduced logistic regression, a method for analyzing binary outcomes with a small number of observations.^31^ We summarized differences using odds ratios and average marginal effects (AME)^32^ and computed *P* values for differences in proportions using Wald tests. The AME for arm (WNP vs control) is the average, taken over the whole cohort, of the patient-level difference between the model’s predicted probability of the outcome (eg, ≥5% or ≥10% weight loss), with the arm set to WNP and other covariates set at each patient’s observed values and the same predicted probability with the arm set to control.

We compared 12-month weight change using a difference-in-differences analysis based on a linear mixed model with patient random effects and exposures of time, arm (WNP or control), and a time × arm interaction. Patients with missing 12-month weight data were excluded from weight change analyses; we assessed differences between patients with and without follow-up weight data. In a sensitivity analysis, we adjusted for variables with an SMD of 0.15 or more in magnitude. Among WNP patients, we conducted exploratory analyses to examine weight change among those who had vs did not have 1 or more visit with a health system WMT and/or an AOM prescription and those who did vs did not report weight via text message. We report regression results with 95% CIs. For all analyses, a 2-sided value of *P* < .05 was considered statistically significant. All analyses were performed using R, version 4.1.3 (R Project for Statistical Computing); the optmatch package was used for the propensity matching.

## Results

Of WNP-eligible patients (N = 1159), 54.6% were female, mean (SD) age was 56.2 (12.9) years, and mean (SD) BMI was 37.2 (6.64) (eTable 2 in Supplement 1). Primary care practitioners referred 253 patients to the WNP; 219 (18.9%) met eligibility criteria and 132 (11.4% of referred; 60.3% of eligible) completed a WNP visit (Figure). Patients who were female, had a higher BMI, and were younger were more likely to be referred to the WNP (eTable 3 in Supplement 1). Patients without T2D (adjusted odds ratio [AOR], 3.15; 95% CI, 1.26-8.12) and those from wealthier neighborhoods (AOR, 3.10; 95% CI, 0.64-5.68) were more likely to complete a WNP visit after referral. At the control site, 2156 WNP-eligible patients had a primary care visit during the evaluation period. Eligible controls were older, had lower baseline weight and BMI, and were less likely to be White and privately insured (eTable 2 in Supplement 1).

**Figure.**
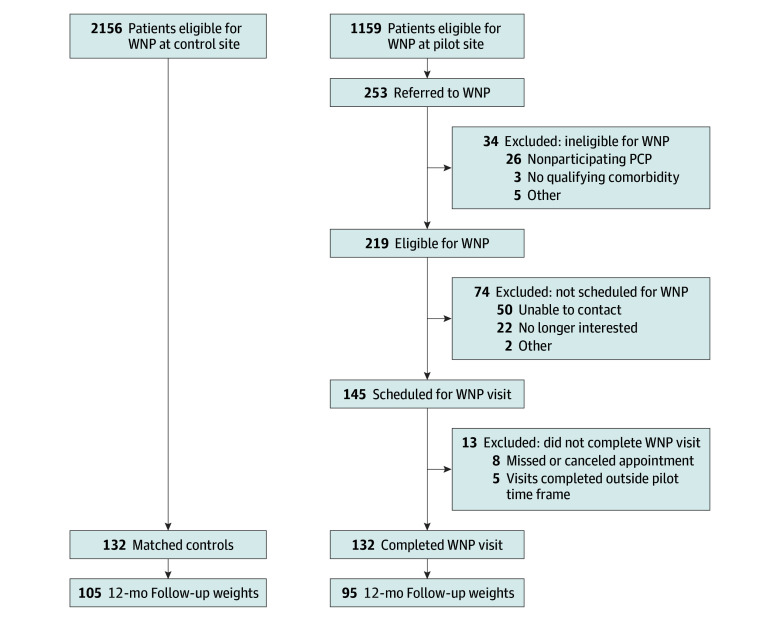
Study Flow Diagram Results of the screening, selection, and analysis processes for the eligible patients at the control and pilot sites. PCP indicates primary care practitioner; WNP indicates weight navigation program.

### Propensity-Matched Results

All WNP participants were successfully matched to controls. The cohorts were well balanced on variables included in the propensity score model, with all SMDs less than or equal to 0.11. Of WNP patients (n = 132) and matched controls (n = 132), 181 (68.6%) were women and 83 (31.4%) were men. Mean (SD) age was 49.5 (13.0) years. Race and ethnicity included African American, 52 (19.7%); Asian or American Indian/Alaska Native, 14 (5.3%); and White, 180 (68.2%) (Table 1). There was some remaining imbalance in diagnosis of hyperlipidemia (SMD, −0.31), Hispanic ethnicity (SMD, 0.15), and Medicaid insurance (SMD, −0.23).

**Table 1.** Baseline Patient Characteristics

Characteristic	No. (%)			*P* value	Standardized mean difference
	WNP patients	Matched controls	Overall		
Patients	132 (100)	132 (100)	264 (100)		
Age, mean (SD), y	49.8 (11.1)	49.2 (14.7)	49.5 (13.0)	.70	−0.05
Sex					
Female	93 (70.5)	88 (66.7)	181 (68.6)	.60	0.08
Male	39 (29.5)	44 (33.3)	83 (31.4)		
Race^a^					
African American	24 (18.2)	28 (21.2)	52 (19.7)	.82	0.07
Asian or American Indian/Alaska Native	8 (6.1)	8 (6.1)	14 (5.3)		0
Caucasian	94 (71.2)	86 (65.2)	180 (68.2)		−0.13
Other	6 (4.5)	8 (6.1)	14 (5.3)		0.07
Missing	0	2 (1.5)	2 (0.8)		
Ethnicity					
Non-Hispanic/Latine	129 (97.7)	125 (94.7)	254 (96.2)	.33	0.15
Hispanic/Latine	3 (2.3)	7 (5.3)	10 (3.8)		
Primary insurance payer					
Medicaid	14 (10.6)	6 (4.5)	20 (7.6)	.15	−0.23
Medicare	15 (11.4)	20 (15.2)	35 (13.3)		0.11
Private insurance	99 (75.0)	99 (75.0)	198 (75.0)		0
Other insurance	4 (3.0)	7 (5.3)	11 (4.2)		0.07
Baseline weight, mean (SD), kg	114 (25.5)	113 (26.3)	114 (25.9)	.75	−0.04
Baseline BMI, mean (SD)	40.6 (8.38)	39.8 (7.93)	40.2 (8.15)	.42	−0.09
Weight-related conditions					
Hyperlipidemia	61 (46.2)	41 (31.1)	102 (38.6)	.44	−0.31
Hypertension	85 (64.4)	90 (68.2)	175 (66.3)		0.08
Metabolic dysfunction–associated steatotic liver disease	27 (20.5)	28 (21.2)	55 (20.8)		0.02
Sleep apnea	81 (61.4)	81 (61.4)	162 (61.4)		0
Type 2 diabetes	31 (23.5)	27 (20.5)	58 (22.0)		−0.07
Total weight-related conditions, mean (SD)	2.16 (1.05)	2.02 (1.12)	2.09 (1.08)	.31	−0.12
Proportion of neighborhood with annual household income>$75 000, mean (SD)	0.58 (0.16)	0.60 (0.19)	0.59 (0.18)	.37	0.11

Abbreviations: BMI, body mass index (calculated as weight in kilograms divided by height in meters squared); WNP, weight navigation program.

^a^ Race categories included in the electronic health record data (based on patient self-identification) were African American, American Indian or Alaska Native, Asian, Native Hawaiian or Other Pacific Islander, White, other, patient refused, and unknown.

In each arm among 132 patients, 95 (72%) WNP patients and 105 (80%) controls had 12-month weight data within a mean (SD) of 25 (24) days and 30 (25) days of baseline plus 12 months. African American patients had lower odds of missing 12-month weight data (OR, 0.23; 95% CI, 0.06-0.79); there were no other statistically significant differences among those with and without 12-month weight data. During the study period, 120 patients (91%) had 1 WNP visit, 10 (8%) had 2 visits, and 2 (2%) had 3 or more visits.

Mean weight change was −5.4 kg (95% CI, −7.8 to 3.0 kg) among WNP patients and −0.3 kg (95% CI, −1.9 to 1.4 kg) among controls (Table 2). Mean percent weight change was −4.4% (95% CI, −6.4% to −2.5%) among WNP patients and −0.1% (95% CI, −1.3% to 1.4%) among controls. In a difference-in-differences analysis, WNP patients lost 4.9 kg more weight than controls (95% CI, 2.11-7.76; *P* < .001) and had 4.4% greater weight loss (95% CI, 2.2%-6.4%; *P* < .001). Patients in the WNP vs control group were more likely to achieve weight loss of 5% or more (41.1% vs 17.9%; OR, 2.90; 95% CI, 1.54-5.58; AME, 21.2%; *P* < .001), and 10% or more (AME: 22.1%; 95% CI, 8.8%-33.6%; vs 3.8%; OR, 7.19; 95% CI, 2.55-25.9; AME: 17.4%; 95% CI, 8.7%-26.2%; *P* < .001). Results were similar when adjusting for unbalanced variables (eTable 4 in Supplement 1). Patients in the WNP who had 1 or more visit with a health system WMT or were prescribed AOMs (45 of 95 [47%]) lost 1.7 kg more than those who did not (−6.3 vs −4.6 kg; *P* = .48). There were no significant differences in weight change among WNP patients who did vs did not report weight via text message (eTable 5 in Supplement 1).

**Table 2.** Within-Group and Between-Group Weight Change Comparisons, Baseline to 12 Months

Variable	Estimate (95% CI)		Unadjusted difference or OR (95% CI)	AOR (95% CI)^a^	Average marginal effects (95% CI)	*P* value for difference or average marginal effects
	WNP patients	Matched controls				
Patients, No. (%)	95 (100)	106 (100)	NA	NA	NA	NA
Mean weight change, kg^b^	−5.4 (−7.8 to 3.0)	−0.3 (−1.9 to 1.4)	−4.9 (−7.76 to −2.11)	NA	NA	<.001
Mean % weight change^b^	−4.4 (−6.4 to −2.5)	−0.1 (−1.3 to 1.4)	−4.4 (−6.77 to −2.17)	NA	NA	<.001
≥5% Weight loss, No. (%)^c^	39 (41.1)	19 (17.9)	2.96 (1.58 to 5.67)	2.90 (1.54 to 5.58)	21.2 (8.8 to 33.6)	<.001
≥10% Weight loss, No. (%)^c^	21 (22.1)	4 (3.8)	7.17 (2.60 to 25.3)	7.19 (2.55 to 25.9)	17.4 (8.7 to 26.2)	<.001

Abbreviations: AOR, adjusted odds ratio; NA, not applicable; OR, odds ratio; WNP, weight navigation program.

^a^ Adjusted for initial weight.

^b^ Difference-in-differences linear regression model.

^c^ Logistic regression model.

Patients in the WNP were more likely than controls to be referred to bariatric surgery (18.9% vs 9.1%; *P* = .02), a very low-calorie meal replacement program (16.7% vs 3.8%; *P* < .001), and a Mediterranean-style eating and physical activity program (10.6% vs 1.5%; *P* = .002) (Table 3). There were no significant between-group differences in rates of nutrition counseling referrals (15.2% vs 11.4%; *P* = .36), diabetes prevention program referrals (0% vs 0.8%; *P* = .32), or AOM prescriptions (14.4% vs 10.6%; *P* = .35). Patients in the WNP were prescribed glucagonlike peptide-1 receptor agonists approved for treatment of T2D at a higher rate than controls (8.3% vs 0%; *P* < .001), with most patients (9 of 11 [81.8%]) having a diagnosis of T2D. Patients in the WNP had higher rates of engagement in bariatric surgery consultation (15.2% vs 6.8%; *P* = .03) and a Mediterranean-style eating and physical activity program (4.5% vs 0%; *P* = .01), along with higher rates of completed bariatric surgeries (3.8% vs 0%; *P* = .02).

**Table 3.** Referral to and Engagement in Weight Management Treatments

Weight management treatment	No. (%)			OR (95% CI)^a^	Difference (95% CI)^b^	*P* value for difference^b^
	WNP patients	Matched controls	Overall			
Patients	132 (100)	132 (100)	264 (100)			
Bariatric surgery						
Referral	25 (18.9)	12 (9.1)	37 (14.0)	2.34 (1.14 to 5.03)	13.4 (3.7 to 23.0)	.02
Engagement^c^	20 (15.2)	9 (6.8)	29 (11.0)	2.44 (1.10 to 5.84)	10.5 (2.1 to 19.0)	.03
Completed surgery	5 (3.8)	0	5 (1.9)	11.43 (0.63 to 208.9)	3.9 (0.6 to 4.3)	.02
Very low-calorie meal replacement program						
Referral	22 (16.7)	5 (3.8)	27 (10.2)	5.08 (2.00 to 15.6)	16.1 (7.9 to 24.3)	<.001
Engagement^c^	10 (7.6)	3 (2.3)	13 (4.9)	3.17 (0.92 to 10.9)	5.9 (0.4 to 11.4)	.05
Mediterranean-style eating and physical activity program						
Referral	14 (10.6)	2 (1.5)	16 (6.1)	6.39 (1.63 to 25.0)	10.3 (4.1 to 16.5)	.002
Engagement^c^	6 (4.5)	0	6 (2.3)	13.62 (0.76 to 244.2)	4.8 (1.0 to 8.5)	.01
Nutrition counseling						
Referral	20 (15.2)	15 (11.4)	35 (13.3)	1.39 (0.68 to 2.90)	5.0 (−4.3 to 14.4)	.36
Engagement^c^	14 (10.6)	7 (5.3)	21 (8.0)	2.12 (0.85 to 5.76)	6.3 (−0.8 to 13.4)	.11
Diabetes prevention program^d^						
Referral	0	1 (0.8)	1 (0.4)	0.33 (0.01 to 8.19)^e^	−0.8 (−2.3 to 0.7)	.32
Antiobesity medications^d^						
Prescription	19 (14.4)	14 (10.6)	33 (12.5)	1.42 (0.68 to 3.01)	4.9 (−4.1 to 14.0)	.35
GLP-1 RAs approved for type 2 diabetes^d^						
Prescription	11 (8.3)^f^	0	11 (4.2)	25.08^e^ (1.46 to 430.2)	9.1 (4.0 to 14.2)	<.001

Abbreviations: GLP-1 RA, glucagonlike peptide-1 receptor agonists; OR, odds ratio; WNP, weight navigation program.

^a^ Reference group is matched cohort.

^b^ Confidence intervals and *P* values for differences were computed using Wald difference in proportions.

^c^ Engagement is defined as having at least 1 completed encounter with the weight management treatment after referral.

^d^ Unable to determine engagement in diabetes prevention programs or use of prescribed medications due to data limitations.

^e^ Odds computed using Firth bias-reduced logistic regression.

^f^ Of 11 patients, 9 (82%) had a type 2 diabetes diagnosis and 2 patients did not.

## Discussion

This study evaluated the feasibility and weight loss outcomes of a pilot WNP consisting of weight-focused visits with an ABOM diplomate and preference-sensitive use of WMT options. During the 12-month evaluation period, 1159 eligible patients had a primary care visit at the WNP site, 219 (18.9%) were referred to the WNP, and 132 (60.3% of referred patients) completed a visit. Although PCPs referred few eligible patients to the WNP, the rate of referral was higher than previously reported rates of PCPs’ referrals to other WMTs, including nutrition counseling services (1%-11%)^33,34^ and bariatric surgery (5%).^35^ Our team’s prior work suggests that PCPs desire support from obesity medicine experts,^12^ and we aim to develop and test strategies (eg, EHR-based alerts and decision-support tools) to augment PCPs’ referral of WNP-eligible patients.

At the end of the 12-month follow-up, WNP patients achieved significantly greater weight loss than controls and had significantly greater odds of achieving 5% or more and 10% or more weight loss (WNP: 2.90 and controls: 7.19). Most WNP patients (91%) required only 1 WNP visit and were referred at significantly higher rates than controls to health system WMTs, including bariatric surgery, a very low-calorie meal replacement program, and a Mediterranean-style eating and activity program. These findings underscore the weight loss effectiveness of the WMTs outside of research that have, to date, been severely underused.^8^

There was no statistically significant difference in AOM prescribing between the WNP and control groups. This may be due, in part, to our inability to fully accommodate PCPs’ request for AOMs to be initiated and titrated to a stable dose by the WNP diplomates due to limited capacity for WNP follow-up visits. Thus, WNP patients preferring AOMs may not have received the medications during the pilot period. We are now refining the WNP to include population health management strategies (eg, EHR-based outreach to patients prescribed AOMs) to support AOM prescribing while minimizing WNP follow-up visits.

The WNP’s preference-sensitive use of all available WMTs is novel compared with other primary care–based obesity treatment interventions. For example, a recent systematic review and meta-analysis of randomized clinical trials of weight management interventions in primary care settings reported predominant use of lifestyle counseling interventions.^36^ While such interventions can support modest weight loss, they generally lack preference sensitivity and may be difficult to sustain due to use of study-specific personnel and other resources not available in most practice settings. Limited prior work has observed the outcomes of weight-focused visits with primary care–based ABOM diplomates^21,37^ and with nondiplomate PCPs assisted by EHR-based tools to support obesity treatment decision-making.^38^

### Limitations

This study has several limitations. First, the WNP was implemented at a single primary care site in 1 large, academic health system, and its results may not generalize to other practice settings. However, its key components, including weight-focused visits with PCPs’ knowledgeable about obesity medicine, may be feasible in diverse primary care settings. Second, the study period largely predated the availability of newer, highly effective incretin mimetics for weight management. The US Food and Drug Administration approval of semaglutide^39^ and tirzepatide^40^ for weight management, coupled with direct-to-consumer marketing approaches,^41^ has heightened patients’ desire for these treatments, and our findings may not reflect current WMT use. We plan to conduct future analyses of patients’ AOM use and weight change among a WNP vs matched controls. Third, patients face differential insurance coverage for WMTs, and we are unable to assess how cost influenced patients’ treatment selection. Fourth, there may be between-group differences in patients’ motivation to lose weight and/or willingness to engage in WMT; these constructs are not captured in the EHR and could not be accounted for through propensity matching.

## Conclusions

In this cohort study of patients referred to a pilot WNP, the program was feasible and associated with greater WMT use and weight loss than observed in matched controls. The WNP is a promising model to improve obesity treatment in primary care settings and warrants rigorous evaluation in a large-scale randomized clinical trial with longer-term assessment of outcomes and determinants of implementation.

## Notes

Supplement 1. **eTable 1.** Odds Ratios for Propensity Model **eTable 2.** Baseline Characteristics of WNP Eligible Patients at Control Site and Weight Navigation Program (WNP) Pilot Site **eTable 3.** Odds Ratios for Referral to WNP Among Patients Eligible for WNP Referral at the Pilot Site **eTable 4.** Within and Between Group Weight Change Comparisons, Baseline to 12 Months, Adjusted for Unbalanced Baseline Variables **eTable 5.** Difference-in-Differences Sensitivity Analysis Among WNP Patients With and Without Weight Reporting via Text Message

Supplement 2.
Data Sharing Statement
