# Supplementary material for: A Primary Care–Based Weight Navigation Program
Source: JAMA Netw Open. 2024 May 21;7(5):e2412192. doi: 10.1001/jamanetworkopen.2024.12192 (PMC11109771; doi:10.1001/jamanetworkopen.2024.12192)
Supplement: Supplement 1. — eTable 1. Odds Ratios for Propensity Model eTable 2. Baseline Characteristics of WNP Eligible Patients at Control Site and Weight Navigation Program (WNP) Pilot Site eTable 3. Odds Ratios for Referral to WNP Among Patients Eligible for WNP Referral at the Pilot Site eTable 4. Within and Between Group Weight Change Comparisons, Baseline to 12 Months, Adjusted for Unbalanced Baseline Variables eTable 5. Difference-in-Differences Sensitivity Analysis Among WNP Patients With and Without Weight Reporting via Text Message [file jamanetwopen-e2412192-s001.pdf]

Supplementary Online Content

Griauzde DH, Turner CD, Othman A, et al. A primary care–based weight navigation program. *JAMA Netw Open*. 2024;7(5):e2412192. doi:10.1001/jamanetworkopen.2024.12192

eTable 1. Odds Ratios for Propensity Model

eTable 2. Baseline Characteristics of WNP Eligible Patients at Control Site and Weight Navigation Program (WNP) Pilot Site

eTable 3. Odds Ratios for Referral to WNP Among Patients Eligible for WNP Referral at the Pilot Site

eTable 4. Within and Between Group Weight Change Comparisons, Baseline to 12 Months, Adjusted for Unbalanced Baseline Variables

eTable 5. Difference-in-Differences Sensitivity Analysis Among WNP Patients With and Without Weight Reporting via Text Message

This supplementary material has been provided by the authors to give readers additional information about their work.

© 2024 Griauzde DH et al. *JAMA Network Open.*

eTable1. Odds Ratios for Propensity Model

This table shows the odds ratios associated with the coefficients for the propensity for referral to the weight navigation program among all eligible patients modeled using multivariate logistic regression.

**Characteristic**	**Odds Ratio (95% CI)**[a]	**p-value**
Age, years	0.98 (0.96-1.00)	0.06
Baseline body mass index (BMI), kg/m^2	1.06 (1.04-1.09)	< 0.001
Female sex	1.89 (1.24-2.91)	0.004
Caucasian race	0.66 (0.35-1.32)	0.20
African American race	0.96 (0.46-2.10)	0.90
Private insurance	1.32 (0.74-2.46)	0.37
Medicare insurance	0.47 (0.21-1.09)	0.08
Hypertension	1.05 (0.69-1.62)	0.86
Type 2 diabetes	0.59 (0.37-0.93)	0.03
Metabolic dysfunction-associated steatotic liver disease (MASLD)	1.69 (1.01-2.74)	0.04
Sleep apnea	1.54 (1.03-2.31)	0.04
Proportion of households in neighborhood with household income >$75K	4.62 (1.51-14.7)	0.008

Abbreviation: CI = Confidence Interval

© 2024 Griauzde DH et al. *JAMA Network Open.*

eTable2. Baseline Characteristics of WNP Eligible Patients at Control Site and Weight Navigation Program (WNP) Pilot Site

Characteristic	Control Site, N (%)	Pilot Site, N (%)	Overall, N (%)	p-value
Patients, N	**2,156**	**1,159**	**3,315**	
Age in years, mean (sd)	58.0 (13.9)	56.2 (12.9)	57.4 (13.6)	< 0.001
Sex				0.86
Female	1186 (55.0)	633 (54.6)	1819 (54.9)	
Male	959 (44.5)	520 (44.9)	1479 (44.6)	
Missing	11 (0.5)	6 (0.5)	17 (0.5)	
Race				0.02
African American	387 (18.0)	181 (15.6)	568 (17.1)	
Asian, American Indian/Alaska Native, Native Hawaiian/Pacific Islander	117 (5.4)	41 (3.5)	158 (4.8)	
Caucasian	1557 (72.2)	876 (75.6)	2433 (73.4)	
Other Race[a]	65 (3.0)	41 (3.5)	106 (3.2)	
Missing	30 (1.4)	33 (2.8)	63 (1.9)	
Ethnicity				0.46
Non-Hispanic/Latine	2072 (96.1)	1120 (96.6)	3192 (96.3)	
Hispanic/Latine	73 (3.4)	33 (2.8)	106 (3.2)	
Missing	11 (0.5)	6 (0.5)	17 (0.5)	
Primary insurance payor				< 0.001
Private Insurance	1019 (47.3)	645 (55.7)	1664 (50.2)	
Medicare	886 (41.1)	382 (33.0)	1268 (38.3)	
Medicaid	161 (7.5)	85 (7.3)	246 (7.4)	
Other Insurance	79 (3.7)	41 (3.5)	120 (3.6)	
Missing	11 (0.5)	6 (0.5)	17 (0.5)	
Baseline weight in kilograms (kg), mean(sd)	104 (20.6)	107 (21.6)	105 (21.0)	< 0.001
Baseline BMI in kg/m^2, mean(sd)	36.4 (6.04)	37.2 (6.64)	36.7 (6.26)	< 0.001
Weight-related conditions				0.76
Hyperlipidemia	1080 (50.1)	582 (50.2)	1662 (50.1)	
Hypertension	1578 (73.2)	818 (70.6)	2396 (72.3)	
Metabolic dysfunction-associated steatotic liver disease (MASLD)	274 (12.7)	159 (13.7)	433 (13.1)	
Sleep Apnea	1052 (48.8)	588 (50.7)	1640 (49.5)	
Type 2 Diabetes	753 (34.9)	410 (35.4)	1163 (35.1)	
Total weight-related conditions, mean(sd)	2.21 (1.08)	2.22 (1.08)	2.21 (1.08)	0.81
Proportion of neighborhood with annual household income >$75K, mean (sd)	0.57 (0.191)	0.56 (0.188)	0.57 (0.190)	0.23

[a] This category includes patients who self-identify as Other Race based on EHR data
Abbreviations: SD = Standard Deviation

© 2024 Griauzde DH et al. *JAMA Network Open.*

eTable3. Odds Ratios for Referral to WNP among Patients Eligible for WNP referral at the Pilot Site

This table shows the odds ratios associated with referral to the weight navigation program among all WBP eligible patients at the Pilot Site modeled using multivariate logistic regression.

**Characteristic**	**Odds Ratio (95% CI)**	**p-value**
Age, years	0.98 (0.97-1.00)	0.044
Baseline body mass index (BMI), kg/m^2	1.07 (1.04-1.10)	< 0.001
Female sex	1.68 (1.16-2.46)	0.007
Caucasian race	0.74 (0.40-1.41)	0.34
African American race	1.13 (0.56-2.33)	0.74
Hispanic ethnicity	0.84 (0.26-2.30)	0.75
Private insurance	1.22 (0.52-3.25)	0.66
Medicare insurance	0.38 (0.14-1.13)	0.07
Medicaid Insurance	1.79 (0.68-5.22)	0.26
Hypertension	0.96 (0.65-1.41)	0.82
Hyperlipidemia	1.08 (0.75-1.56)	0.66
Type 2 diabetes	0.70 (0.47-1.05)	0.09
Metabolic dysfunction-associated steatotic liver disease (MASLD)	1.43 (0.89-2.30)	0.13
Sleep apnea	1.36 (0.95-1.96)	0.10
Proportion of households in neighborhood with household income >$75K	2.28 (0.17-1.84)	0.11

Abbreviations: CI = Confidence Interval, WNP = Weight Navigation Program

© 2024 Griauzde DH et al. *JAMA Network Open.*

eTable4. Within and between group weight change comparisons, baseline to 12 months, adjusted for unbalanced baseline variables.

This table shows a sensitivity analysis of the 12-month weight outcome comparison among Weight Navigation Program (WNP) patients and Matched controls, adjusting for unbalanced baseline variables (defined as a standardized mean difference greater than or equal to the absolute value of 0.15).

Outcome	WNP Patients, estimate (95% CI)	Matched Controls, estimate (95% CI)	Adjusted Difference or Odds Ratio [a] (95% CI)	Average Marginal Effect (%) (95% CI)	p-value for Difference or Average Marginal Effects
Mean weight change[b,] kg	-5.4 (-7.8 to -3.0)	-0.3 (-1.9 to 1.4)	-4.9 (-7.8 to -2.1)	-	< 0.001
Mean % weight change [c]	-4.4 (-6.4 to -2.5)	-0.1 (-1.3 to 1.4)	-4.3 (-6.6 to -2.0)	-	< 0.001
≥ 5% weight loss [d], n (%)	39 (41.1)	19 (17.9)	3.20 (1.7-6.4)	21.9 (9.7-34.0)	< 0.001

[a] Adjusted for initial weights and unbalanced variables, which included Medicaid insurance, presence of hyperlipidemia, and Hispanic ethnicity.
[b] Difference-in-Differences linear regression model for weight.
[c] Difference-in-Differences linear regression model for log weight, transformed to percent change scale.
[d] Logistic Regression Model.

© 2024 Griauzde DH et al. *JAMA Network Open.*

eTable5. Difference-in-Differences Sensitivity Analysis among WNP Patients with and without Weight Reporting via Text Message

This table compares the mean weight change and odds of ≥ 5% weight loss[b] among WNP patients who reported weight via text message and those who participated in WNP without weight reporting via text message.

Outcome	WNP Only vs WNP + Weight Reporting via Text Message, Adjusted Difference or Odds Ratio (95% CI)	p-value
Mean weight change[a,] kg	-0.8 (-1.94 to 1.67)	0.49
≥ 5% weight loss[b], odds ratio	1.67 (0.61-4.99)	0.34

[a] Difference-in-Differences linear regression model for weight, adjusted for initial weight.
[b] Logistic Regression Model.
[c] Reference group is WNP Only.
Abbreviations: CI = Confidence Interval, WNP = Weight Navigation Program

© 2024 Griauzde DH et al. *JAMA Network Open.*
