# Supplementary material for: A Primary Care–Based Weight Navigation Program
Source: JAMA Netw Open. 2024 May 21;7(5):e2412192. doi: 10.1001/jamanetworkopen.2024.12192 (PMC11109771; doi:10.1001/jamanetworkopen.2024.12192)
Supplement: Supplement 2. — Data Sharing Statement [file jamanetwopen-e2412192-s002.pdf]

Data Sharing Statement

Griauzde. A Primary Care–Based Weight Navigation Program. *JAMA Netw Open*. Published May 21, 2024. doi:10.1001/jamanetworkopen.2024.12192

Data
Data available: No
